# The Domestication of the Amazon Tree Grape (*Pourouma cecropiifolia*) Under an Ecological Lens

**DOI:** 10.3389/fpls.2018.00203

**Published:** 2018-03-14

**Authors:** Hermísia C. Pedrosa, Charles R. Clement, Juliana Schietti

**Affiliations:** ^1^Programa de Pós-Graduação em Ecologia, Instituto Nacional de Pesquisas da Amazônia, Manaus, Brazil; ^2^Coordenação de Tecnologia e Inovação, Instituto Nacional de Pesquisas da Amazônia, Manaus, Brazil

**Keywords:** allometry, Amazonia, domestication syndrome, ecological perspective, environmental effects, perennial fruit crop

## Abstract

Domestication studies traditionally focus on the differences in morphological characteristics between wild and domesticated populations that are under direct selection, the components of the domestication syndrome. Here, we consider that other aspects can be modified, because of the interdependence between plant characteristics and the forces of natural selection. We investigated the ongoing domestication of *Pourouma cecropiifolia* populations cultivated by the Ticuna people in Western Amazonia, using traditional and ecological approaches. We compared fruit characteristics between wild and domesticated populations to quantify the direct effects of domestication. To examine the characteristics that are not under direct selection and the correlated effects of human selection and natural selection, we investigated the differences in vegetative characteristics, changes in seed:fruit allometric relations and the relations of these characteristics with variation in environmental conditions summarized in a principal component analysis. Domestication generated great changes in fruit characteristics, as expected in fruit crops. The fruits of domesticated plants had 20× greater mass and twice as much edible pulp as wild fruits. The plant height:DBH ratio and wood density were, respectively, 42% and 22% smaller in domesticated populations, probably in response to greater luminosity and higher sand content of the cultivated landscapes. Seed:fruit allometry was modified by domestication: although domesticated plants have heavier seeds, the domesticated fruits have proportionally (46%) smaller seed mass compared to wild fruits. The high light availability and poor soils of cultivated landscapes may have contributed to seed mass reduction, while human selection promoted seed mass increase in correlation with fruit mass increase. These contrasting effects generated a proportionately smaller increase in seed mass in domesticated plants. In this study, it was not possible to clearly dissociate the environmental effects from the domestication effects in changes in morphological characteristics, because the environmental conditions were intensively modified by human management, showing that plant domestication is intrinsically related to landscape domestication. Our results suggest that evaluation of environmental conditions together with human selection on domesticated phenotypes provide a better understanding of the changes generated by domestication in plants.

## Introduction

Plant domestication resulted in populations more useful to humans and better adapted to cultivated landscapes (Harlan, 1992; Clement, 1999). Although humans have domesticated populations of many species, currently only 12 annual crops account for 75% of world food consumption (FAO, 2004). In the tropics, however, many fruit trees were domesticated in different degrees (Miller and Gross, 2011; Meyer et al., 2012), and in the Amazonia, most species with domesticated populations are fruit trees (Clement, 1999). One of these species is the Amazon tree grape (*Pourouma cecropiifolia*, Urticaceae), cultivated principally in Western Amazonia.

Gonzalo Jiménez de Quesada, in his 1596 expedition in the Eastern Llanos of Colombia, reported the existence of plantations of *P. cecropiifolia* in “*gardens of vegetables and fruit plants*” (Patiño, 2002). He describes the trees with “*large racemes and fruits as large as nuts*” (Patiño, 2002), indicating that the species is cultivated and probably domesticated since the pre-Columbian period. Currently, *P. cecropiifolia* is especially popular among the Ticuna people and large plantations are found in swiddens and fallows around their villages, in the vicinity of Tabatinga (Brazil), Letícia (Colombia), and Iquitos (Peru), where Clement (1989) observed several morphological differences in the fruits between wild and domesticated plants. In this region, the Ticuna people are still selecting individuals of *P. cecropiifolia* that have the largest and sweetest fruits, and removing the individuals that have smaller and tasteless fruits. Annually, in the fruiting period, the Ticuna farmers select and propagate the seeds of the plants with interesting characteristics in cultivated environments.

Because the species is dioecious (Clement, 1989), only fruiting trees can be selected and open-pollination from unselected pollen-donors (and individuals that will be removed later) slows response-to-selection. Like any crop, *P. cecropiifolia* is cultivated in agroecosystems that are quite different from it wild niche in early and mid-successional forests, so there is unconscious human selection for adaptation to new ecological factors. Therefore, *P. cecropiifolia* domestication is a special case compared to annual crops that are often under high intensity selection, because it is a moderately long-lived species, whose domestication syndrome has been influenced by both human selection and changes in ecological conditions that began a long time ago. However, this is a common case in Amazonia, where perennial plants and landscape domestication occur at the same time (Clement, 1999). Considering these points, it is an open question how wild and domesticated populations of perennial plants, such as *P. cecropiifolia*, differ and what are the effects of human selection, environmental conditions, and their interactions on fruits, seeds, and vegetative characteristics.

Domestication studies traditionally approach the differences and variation in morphological and genetic aspects between wild and domesticated plant populations (Harlan, 1992; Clement, 1999; Miller and Gross, 2011). However, a focus only on domesticated characteristics can limit the understanding of the interaction between human selection and natural selection. Because natural selective forces act on the phenotypes along with human selection, the set of characteristics that marks the divergence between domesticated and wild plants, the domestication syndrome, is probably more diverse than understood in classic domestication studies (Milla et al., 2015; Preece et al., 2017). A look at the ecological mechanisms that continue to act during the domestication process considers characteristics that are and are not under human selection and the correlations between characteristics. An ecological approach also allows the identification of relations between morphological characteristics and environment conditions, and evaluation of the integrated effect of domestication and the environment on phenotypic plasticity of the characteristics directly or indirectly modified by human selection. Therefore, considering ecological aspects can generate a more complete and integrated understanding of the domestication process (Milla et al., 2015).

In trees, the domestication process begins with population management in their natural environment (Rindos, 1984; Smith, 2011). Subsequently, individuals with the most desirable morphological characteristics are selected and cultivated in domesticated landscapes (Clement, 1999; Smith, 2011; Levis et al., 2017). Growing conditions under cultivation and directional selection lead the domesticated plant populations to diverge morphologically and genetically from their wild progenitors (Pickersgill, 2007; Miller and Gross, 2011). The genetic variability of populations under selection is reduced due to founder events (Nei et al., 1975) caused by the selection of a few individuals and a restricted gene pool in the next generation (Miller and Gross, 2011). In contrast, the phenotypic variability of the characteristics under selection in domesticated populations may increase in comparison with wild populations (Clement, 1999; Meyer and Purugganan, 2013). In perennial fruit crops, like *P. cecropiifolia*, the average time expected from the beginning of selection to domestication, when the domesticated characteristics are fixed in the cultivated populations, is about 3,000 years (Meyer et al., 2012).

Changes in the morphology of aerial vegetative parts, fruits and seeds are among the most common characteristics of the plant domestication syndrome (Meyer et al., 2012). In herbaceous plants, increases in leaf and whole-plant size are observed (Milla and Morente-López, 2014). In fruit trees, fruit and seed “gigantism” is very frequent (Meyer et al., 2012). These changes in the sizes of useful parts and in the whole-plant occur due to changes in biomass allocation patterns in and among the parts under selection (Milla et al., 2015). The increase in certain plant organs or parts caused by domestication can lead indirectly to changes in size of other plant parts due to allometric or biophysical relationships. In this case, any increase in allocation to an organ should be complemented by a proportional increase to the other organ or it has a cost to the other organ (Kleyer and Minden, 2015). Allometric relationships were analyzed in five herbaceous species and it was observed that plants selected to have larger leaf areas invested less in leaf blade biomass, but invested in larger petioles and other supporting structures, leading to larger plant sizes (Milla and Matesanz, 2017). For fruits and seeds, some allometric relationships are well known; for example, plants that have fruits with larger seeds have a smaller number of seeds per fruit (Niklas, 1994). However, to our knowledge, there are no studies about the allometric relationships in fruit trees to explain the fruit and seed size changes generated by domestication. The changes in fruit and seed allometric relationships might be a signature of fruit tree domestication, and, if observed for several species, can be a parameter to identify fruit species domestication in future research.

In addition to human selection, the environmental conditions where domesticated plants develop also generate selective pressures that affect their phenotypes (Rindos, 1984; Harlan, 1992). When plants are cultivated, humans modify the landscape to create environmental conditions that reinforce the characteristics of interest and that favor the harvesting process. Farmers typically change soil fertility, light availability and reduce competition through the thinning of neighboring plants (Harlan, 1992; Clement, 1999). For example, the increase in available light and in soil fertility can generate an increase in the size of fruits, contributing to human interest. This can also affect other characteristics that are not under human selection, such as characteristics of leaves and roots, wood density and plant height, which respond to soil and light conditions and can be modified indirectly by domestication due to changes in the environmental conditions caused by human management in the landscape. Knowledge of local environmental conditions will help us to evaluate whether they are favorable, unfavorable or do not interfere with human selection. This will help us to understand what is a direct result of domestication and what is a reflection of the interaction between human selection and environmental variations, which results in a phenotypic plasticity response (Schlichting and Levin, 1986; Scheiner, 1993; Gratani, 2014). In the case of *P. cecropiifolia*, the plants are cultivated principally in *terra-firme* swiddens and fallows, where the Ticuna people practice “slash and burn” agriculture that dramatically modifies ecological conditions, especially soil fertility and light intensity (Clement, 1999; Jacovak et al., 2015).

In this study, we investigated the domestication process of *P. cecropiifolia* populations in Western Amazonia, where cultivated populations with large fruits occur in the vicinity of wild populations in the Amazonian forest. We use the traditional approach (focused on differences and variation in morphological characteristics under selection) allied with an ecological approach (human selection effects on allometry and the relations of morphological characteristics with the environment) to answer the following questions: (i) do domesticated populations have distinct morphological characteristics in contrast to wild populations located in adjacent forests and, if so, what is the magnitude of these differences? (ii) has human selection increased the phenotypic variability of characteristics under selection in domesticated populations? (iii) has human selection altered fruit and seed allometric relationships in domesticated plants? and (iv) what is the importance of environmental conditions in explaining the variation in morphological characteristics in wild and domesticated populations?

## Materials and Methods

### Study Area

**FIGURE 1:**
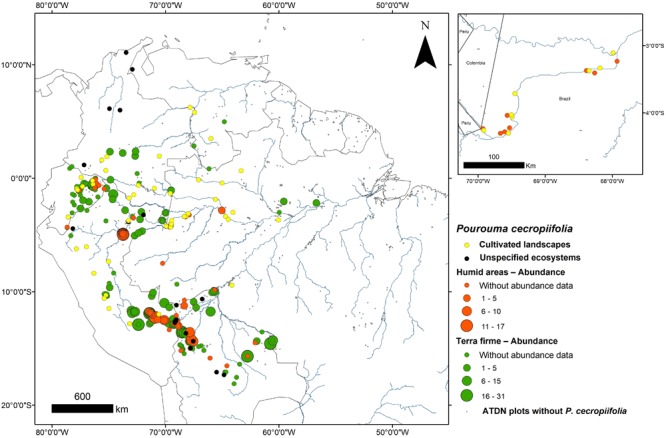
*Pourouma cecropiifolia* distribution and abundance in Greater Amazonia, and study area. Large map: abundance in humid areas (orange) and upland *terra-firme* areas (green) from the Amazon Tree Diversity Network (data provided especially for this paper by the Dr. Hans ter Steege, responsible of the Amazon Tree Diversity Network – ATDN, contact: http://atdn.myspecies.info/); gray dots are ATDN sites with no *P. cecropiifolia*. Occurrence in unspecified ecosystems (black) and cultivated systems (yellow) without abundance data from Missouri Botanic Garden (MOBOT) database (data available in http://tropicos.org/Name/21300486?tab=specimens). Small map: the region of the upper Solimões River with paired cultivated (yellow) and humid area (orange) populations.

This study was conducted in eight Ticuna indigenous communities, along approximately 400 km of the upper Solimões River in Western Brazilian Amazonia (**Figure 1** and **Table 1**). The Ticuna people are the largest indigenous group in Brazil and are distributed in three countries: Brazil, Colombia, and Peru. In Brazil, their communities are located in the state of Amazonas and are distributed along both margins of the Solimões River and its tributaries, where our sampling was performed. The upper Solimões River was chosen as the study area because it has a high concentration of cultivated *P. cecropiifolia* populations and is considered to be the center of domestication (Clement, 1989; Clement et al., 2010). In this region, cultivated populations occur in *terra-firme* areas and wild populations occur in adjacent floodplain forests.

**Table 1:** Data of *Pourouma cecropiifolia* collection sites: population, group (wild, domesticated), village name, city, latitude and longitude.

Population	Group	Village	City	Latitude	Longitude
Pop1	Wild	Umariaçú	Tabatinga-AM	–4.302899	–69.629657
Pop2	Wild	Feijoal	Benjamin Constant-AM	–4.280075	–69.559837
Pop3	Wild	Ourique	Tabatinga-AM	–4.221143	–69.497459
Pop4	Wild	Belém do Solimões	Tabatinga-AM	–4.059635	–69.489769
Pop5	Wild	Vendaval	São Paulo de Olivença-AM	–3.763386	–69.424958
Pop6	Wild	São Francisco do Canimari	Amaturá-AM	–3.370624	–68.349098
Pop7	Wild	Bom Pastor	Amaturá-AM	–3.331287	–68.179170
Pop8	Wild	Lago Grande	Santo Antônio do Içá-AM	–3.23068	–67.892678
Pop9	Domesticated	Umariaçú	Tabatinga-AM	–4.258134	–69.912832
Pop10	Domesticated	Feijoal	Benjamin Constant-AM	–4.300497	–69.545422
Pop11	Domesticated	Ourique	Tabatinga-AM	–4.037632	–69.528462
Pop12	Domesticated	Belém do Solimões	Tabatinga-AM	–4.026873	–69.500669
Pop13	Domesticated	Vendaval	São Paulo de Olivença-AM	–3.744504	–69.480586
Pop14	Domesticated	São Francisco do Canimari	Amaturá-AM	–3.394355	–68.354718
Pop15	Domesticated	Bom Pastor	Amaturá-AM	–3.465094	–68.229006
Pop16	Domesticated	Lago Grande	Santo Antônio do Içá-AM	–3.105835	–67.987486

### Pourouma cecropiifolia

*Pourouma cecropiifolia* is a fruit tree species that occurs in the Amazon rainforest, from Western to Central Amazonia. The species is found in wild conditions in Bolivia, Colombia, Ecuador, Venezuela, Peru, and Brazil. In Brazil, it occurs in the state of Acre and in the state of Amazonas. In areas of primary forest, it occurs mainly in *terra-firme* forests, but abundance in this phyto-physiognomy varies within the distribution area of the species. In the region of upper Solimões River (near the city of Tabatinga, Amazonas, Brazil), for example, it occurs mainly in floodplains, being scarce in *terra-firme* forests. These floodplains are relatively high floodplains, which are flooded for a period of 2–3 months, where the maximum level of flooding is around 1.5 m (personal observation in the year 2015). The occurrence of *P. cecropiifolia* was not recorded in low floodplains or in *chavascais* (almost permanently flooded areas), where there is a great abundance of pioneers, such as species of the genus *Cecropia* (also of the family Urticaceae) and grasses.

The fruiting of the species occurs annually between September and December (Lopes et al., 1999). The main pollinator agents of *P. cecropiifolia* are insects of the family Apidae, *Oxytrigona obscura, Trigona dellatarreana*, and *Trigona* sp. (Falcão and Lleras, 1980). The seed dispersing agents are mainly small-sized primates, bats, and humans. It is considered a fruit of easy propagation, fast growth, precocity and good productivity. Villachica (1996) reports that, in plantations, the trees begin to bear fruit at 2 years, reaching an optimum of production between the fifth and sixth year, with subsequent progressive decrease.

For the Ticuna people, *P. cecropiifolia* is an important traditional fruit and a symbolic component of their culture, and is widely consumed and cultivated in Ticuna fields and agroforests. Moreover, it is reported in Ticuna myths as a plant associated with fauna and mythical entities of the forest.

### Sampling Design and Characteristic Measurements

In each of the eight communities, we sampled 10 adult plants in a *terra-firme* area under cultivation (domesticated population) and 10 adult plants in a nearby forested area (wild population); 3 km was the average distance between paired populations. The paired sampling model follows Dawson et al. (2008), who studied domestication of *Inga edulis* in Western Amazonia. For each individual plant we measured the following morphological characteristics: (i) number of fruits per bunch (mean of five bunches), (ii) fruit length (cm), (iii) fruit diameter (cm), (iv) fruit mass (g), (v) seed mass (g), (vi) peel mass (g), (vii) pulp mass (g) – by difference (iv – v – vi), (viii) pulp:fruit mass ratio – ratio vii:iv, (ix) seed:fruit mass ratio – ratio v:iv, (x) diameter at breast height (DBH) (cm), (xi) plant height (m), to determinate the (xii) plant height:DBH ratio (m/cm) and (xiii) branch wood density (g/cm^3^) (correlation among characteristics are showed in Supplementary Figure S2). For fruit and seed metrics, we used two fruits from each of five bunches. DBH was measured at 1.30 m above ground level. Plant height was estimated using a hypsometer (Vertex Laser VL400 Ultrasonic-Laser Hypsometer III, Haglöf of Sweden). Wood density was determined by the ratio between the dry weight and wet volume of a lateral terminal branch section, with approximately two centimeters in diameter. The wood samples had the wet volume measured by water displacement and were dried for 72 h at 105°C.

### Environmental Conditions

We collected 300-g soil samples in the 0–30 cm layer close to each tree sampled. The 10 individual samples from each population were dried, homogenized and mixed in the laboratory to make a composite sample that represented the soil of each population. The composite sample was analyzed to evaluate phosphorus (P), potassium (K), calcium (Ca), and magnesium (Mg) concentrations (EMBRAPA, 1997). The clay, sand and silt content were determined by granulometric analysis to characterize soil texture (EMBRAPA, 1997).

Light availability was estimated for each tree and averaged over the population. We used the Crown Illumination Index, which describes the environment luminosity inside the forest, on a scale ranging from 1, where there is diffuse incident light, up to 4, where there is direct light on the canopy (Keeling and Phillips, 2007).

### Statistical Analyses

To evaluate the morphological differences between domesticated and wild individuals, we compared the means and amplitudes of variation of 10 characteristics using Kernel density graphs and performed an ANOVA between the two groups (wild and domesticated) for each characteristic, using R software (R Core Team, 2016). A principal component analysis (PCA) of the 10 morphological characteristics was also used to evaluate the differentiation between wild and domesticated individuals, and to evaluate which characteristics are most strongly correlated with domestication, also using R. To test the multivariate differences between wild and domesticated individuals, we performed an ANOVA on the two principal axis of the PCA. To identify and classify groups of wild and domesticated populations, we performed a cluster analysis based on Normal Mixture Modeling, performed with the *mclust* package (Fraley and Raftery, 2002; Fraley et al., 2012) in R. We also identified which group has the greatest phenotypic variability by comparing the variances between the wild and domesticated populations for each characteristic.

To evaluate whether domestication altered allometric patterns of fruit components, we used data from the literature of 74 non-domesticated species with drupe fruits like those of *P. cecropiifolia* (Ibarra-Manriquez and Oyama, 1992; Chen et al., 2010; Shiels and Drake, 2011; Bentos et al., 2012). We adjusted Niklas (1994) potential regression model (SM = a FMˆ^b^) of the relationship between fruit mass (FM) and seed mass (SM) for these non-domesticated species (including non-domesticated *P. cecropiifolia* populations), and used this model for domesticated *P. cecropiifolia* individuals (*n* = 80), performed in the *qpcR* package (Spiess, 2014) in R. We then compared their *shape factors* (a, exponent of the potential relation between variables) and *scaling factors* (b, intercept of the potential relation between variables). The differences between the two equations were evaluated by the overlap of the confidence intervals of the shape and scaling values. We performed a covariance analysis to test the differences in the relations between seed mass and fruit mass considering the three groups – wild individuals of *P. cecropiifolia*, domesticated individuals of *P. cecropiifolia* and the other species.

A PCA of the environmental conditions [described above, plus the sum of bases (K + Ca + Mg), which represents a fertility index] was used to evaluate the differences between the environmental conditions of the forest and cultivated sites. To compare the multivariate differences between forest and cultivated sites, we performed an ANOVA on the first two principal axis of the PCA. We evaluate the effect of environmental conditions on the 10 morphological characteristics through simple linear regressions using all populations together to encompass all the environmental and morphological variation observed in the study. We used the PCA axis that best represented the environmental conditions to evaluate their relationships with morphological characteristics. To evaluate the individual effect of environmental variables, we performed simple linear regressions between each environmental variable and each morphological characteristic. All analyses were run in R.

Finally, we constructed a conceptual model to present an overview of the combined direct and indirect effects of domestication and environmental conditions on the plant phenotype.

## Results

### Morphometry and Domestication Syndrome

**FIGURE 2:**
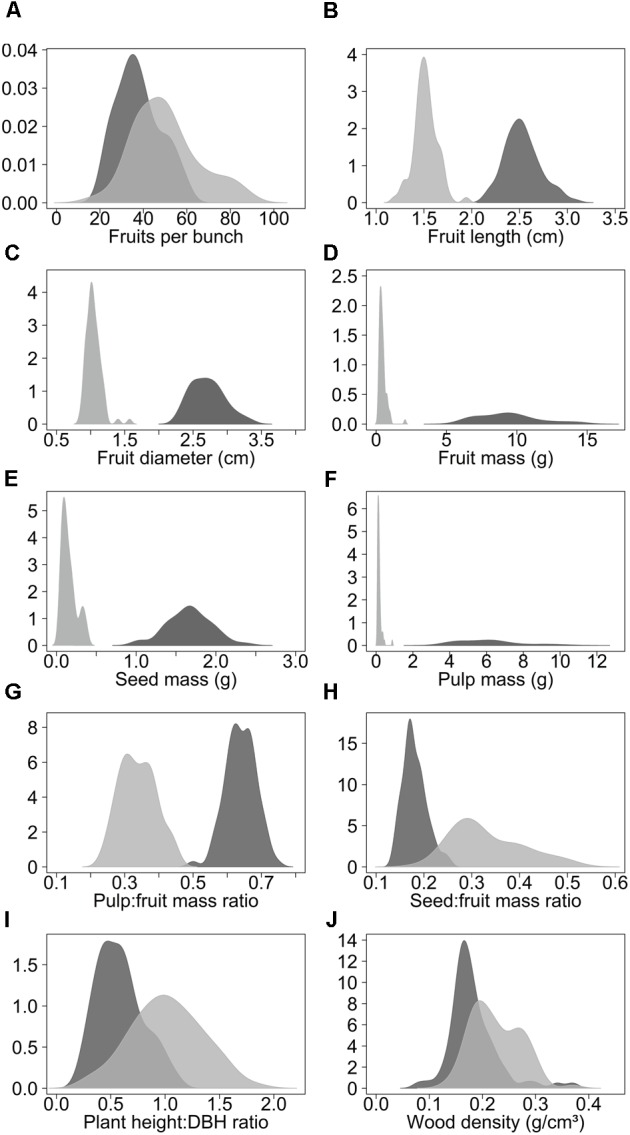
Density curves of the variation of the morphological characteristics of domesticated (dark gray) and wild populations (light gray) of *Pourouma cecropiifolia*. Domesticated populations (*n* = 8, 80 individuals) were collected in cultivated landscapes and wild populations (*n* = 8, 80 individuals) were collected in adjacent floodplain forests, both along the upper Solimões River, Amazonas, Brazil.

Domestication increased the length, diameter and mass of fruits, seed mass, pulp mass and pulp:fruit mass ratio. In contrast, domestication reduced the number of fruits per bunch, seed:fruit mass ratio (seed:fruit allometry), plant height:DBH ratio and wood density (**Figure 2, Table 2**, and **Supplementary Table S1**). Domesticated fruits had 20× greater mass than wild fruits. About 64% of the domesticated fruit is composed of edible pulp, compared to only 34% in wild fruits (**Table 2** and **Supplementary Table S1**). On the other hand, the average values of fruits per bunch, seed:fruit mass ratio, plant height:DBH ratio and wood density were 24.8%, 45.5%, 42.1%, and 21.7% higher in wild populations, respectively (**Table 2** and **Supplementary Table S1**). We found significant differences (*p* < 0.01) between wild and domesticated populations for all 10 characteristics evaluated (**Supplementary Table S2**).

**Table 2:** Values of means and variances per plant group (domesticated and wild) of the 10 morphological characteristics evaluated in the study for *Pourouma cecropiifolia*.

Trait	Wild		Domesticated	
	mean	Variance	mean	variance
Fruits per bunch	50.30	**97.9369**	37.82	48.3784
Fruit length (cm)	1.52	0.0048	2.53	**0.0137**
Fruit diameter (cm)	1.04	0.0060	2.71	**0.0274**
Fruit mass (g)	0.45	0.0343	9.41	**2.6361**
Seed mass (g)	0.16	0.0053	1.67	**0.0226**
Pulp mass (g)	0.17	0.0050	6.11	**1.9019**
Pulp:fruit mass ratio	0.34	0.0008	0.64	**0.0012**
Seed:fruit mass ratio	0.33	**0.0027**	0.18	0.0003
Plant height: DBH ratio (m/cm)	1.02	**0.0145**	0.59	0.0097
Wood density (g/cm^3^)	0.23	0.0003	0.18	**0.0005**

*The bolder values indicate the higher variance between the wild and domesticated group for each characteristic.*

**FIGURE 3:**
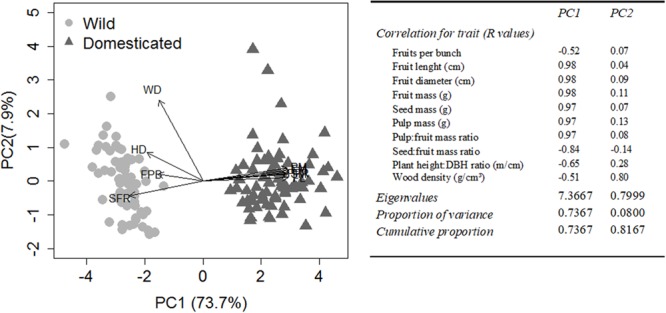
Principal component analysis (PCA) of 10 morphological characteristics of *Pourouma cecropiifolia*, both wild and domesticated plants (*n* = 160). Left, a bivariate plot showing the distribution of individual trees in the multivariate space of morphological characteristics. Right, the correlation between the morphological characteristics and the first two axes of PCA (PC1 and PC2), the eigenvalues and the proportions of variance explained by each PCA axis. FPB, fruits per bunch; FL, fruit length; FD, fruit diameter; FM, fruit mass; SM, seed mass; PM, pulp mass; PFR, pulp:fruit mass ratio; SFR, seed:fruit mass ratio; HD, plant height:DBH ratio; WD, wood density.

The first axis of the PCA with the 10 morphological characteristics explained 73.7% of the data variation highlighting the multivariate differences between wild and domesticated plants (*F* = 1794, *p* < 0.001). The second axis explained 7.9% of the data variation and did not differentiate wild from domesticated plants (*F* = 0.394, *p* = 0.531) (**Figure 3**). The characteristics most associated with the domestication syndrome, mass, proportion and size of fruits and their components (seed and pulp), were highly and positively correlated (±90%) with axis 1. Hence, PC1 is the axis that best reflects the domestication syndrome.

**FIGURE 4:**
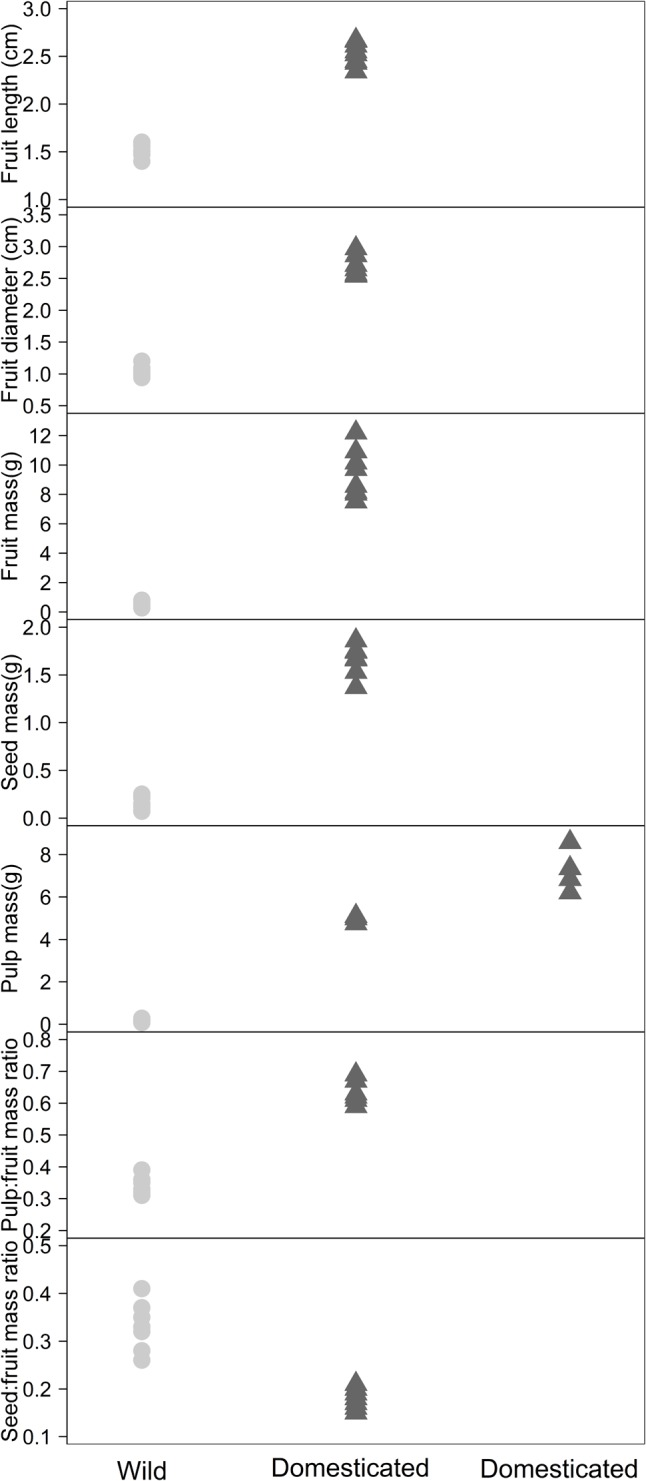
Separation of *Pourouma cecropiifolia* populations in groups of wild and domesticated plants according the mean and variance of seven morphological characteristics evaluated in this study, using cluster discrimination from Normal Misture Modeling.

Reinforcing the pattern found in the PCA, the clustering and classification analysis (Normal Mixture Modeling) distinguished among groups of domesticated and wild populations for seven morphological characteristics (**Figure 4**). Fruit length, fruit diameter, fruit mass, seed mass, pulp:fruit mass ratio and seed:fruit mass ratio discriminated two groups, the domesticated populations and the wild populations. Pulp mass, however, allowed discrimination of three groups, dividing the domesticated populations into two groups, including four populations in and close to Tabatinga, with higher values of pulp mass than the other four populations further east in the study area. Using the clustering and classification analyses, the number of fruits per bunch, plant height:DBH ratio and wood density did not differentiate wild populations from domesticated populations.

### Phenotypic Variability of Characteristics

Among the fruit characteristics, fruit length, fruit diameter, fruit mass, seed mass, pulp mass, and pulp:fruit mass ratio presented higher variances in domesticated populations, indicating greater phenotypic variability in these characteristics in plants under human selection (**Table 2**). Fruit mass and pulp mass presented much greater variances within the domesticated group. The greater amplitude of variation in these characteristics is also apparent in the density curves (**Figure 2**). Number of fruits per bunch, seed:fruit mass ratio and plant height:DBH ratio presented higher variances in wild populations, while wood density presented similar variances in wild and domesticated populations.

### Allometric Changes in Domesticated Plants

**FIGURE 5:**
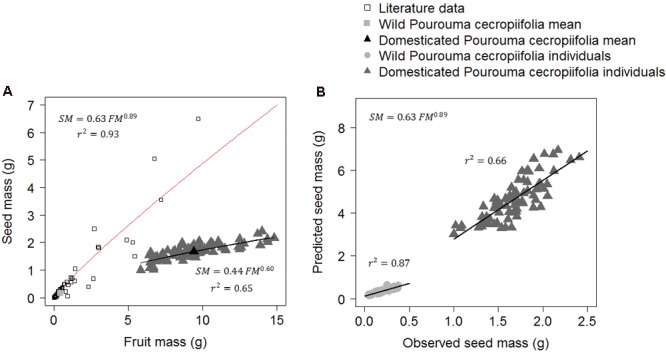
Allometric relations between seed mass (SM) and fruit mass (FM) in fleshy fruits with only one seed (drupes) in 74 wild species and in domesticated *Pourouma cecropiifolia*. **(A)** The red curve represents the adjusted equation of Niklas (1994) using the mean values of 74 non-domesticated species (data compiled from the literature) and the mean value of wild *P. cecropiifolia* (gray square). The black curve represents the equation using fruit and seed mass values of domesticated *P. cecropiifolia* individuals (*n* = 80). **(B)** Correlations between the values predicted by the general allometric equation of wild species **(A)** and the observed seed mass values of wild and domesticated *P. cecropiifolia* individuals.

The general allometric model SM = 0.63 FM^0.89^ (where SM is seed mass and FM is fruit mass) based on 74 species that have fleshy fruits with only one seed (including *P. cecropiifolia* wild populations) presented a higher value of the *shape factor* than the model adjusted to the characteristics of domesticated individuals (SM = 0.44 FM^0.60^). There was no difference in the confidence intervals of the *scaling factor* between the equations. In the equation for domesticated individuals the confidence interval of the *scaling factor* ranged from 0.35 to 0.55 and in the general equation for 74 wild species it ranged from 0.52 to 0.74. The confidence intervals of the *shape factor* values of the equations did not overlap. In the equation of domesticated individuals, the confidence interval of the *shape factor* ranged from 0.50 to 0.69 and in the general equation for 74 wild species it ranged from 0.83 to 0.96. This shows that the two equations are different and that the observed values of seed mass in the *P. cecropiifolia* domesticated plants are lower than the values predicted by the general allometric equation. The seed:fruit mass ratio changed from approximately 0.9:1 in wild plants to 0.6:1 in domesticated plants (**Figure 5A**). In comparison, the correlation between the observed seed mass and the predicted seed mass by the model is higher in wild *P. cecropiifolia* plants (*r*^2^ = 0.87) than in domesticated plants (*r*^2^ = 0.66, **Figure 5B**). In the ANCOVA, we found significant differences in the intercept and in the slopes between the groups of other species, the wild individuals of *P. cecropiifolia* and the domesticated individuals of *P. cecropiifolia* [*F*(2,215) = 267.14, *p* < 0.001]. The interaction between fruit mass and groups was also significant [*F*(2,215) = 228.33, *p* < 0.001], showing that the allometric relation between seed mass and fruit mass change as a function of the groups.

### Effects of Environmental Conditions on Characteristics

The environmental conditions where the wild and the domesticated groups occur in the study area were also very different from each other (**Supplementary Figure S1** and **Supplementary Table S3**). The first principle component explained 91.9% of the data variation, and differentiated floodplain forests from cultivated sites [*F*(1,14) = 18.77, *p* < 0.001]. The second axis explained 7.3% and did not differentiate floodplain forests from cultivated sites (*F* = 3.128, *p* = 0.098). Domestication is strongly correlated with variations in light availability (CII), sum of bases, and calcium, magnesium, phosphorus, silt and sand (**Supplementary Figure S3**). Cultivated sites (*terra-firme*) have 28% higher light availability, poorer soils (16x lower sum of bases), and 63% sandier soils than floodplain forests (right side of **Supplementary Figure S1**); the floodplain forest sites have lower light availability and more fertile silty soils (**Supplementary Table S3**). Only the clay and potassium contents were slightly altered in cultivated areas and are less correlated with domestication (**Supplementary Figure S3**).

**FIGURE 6:**
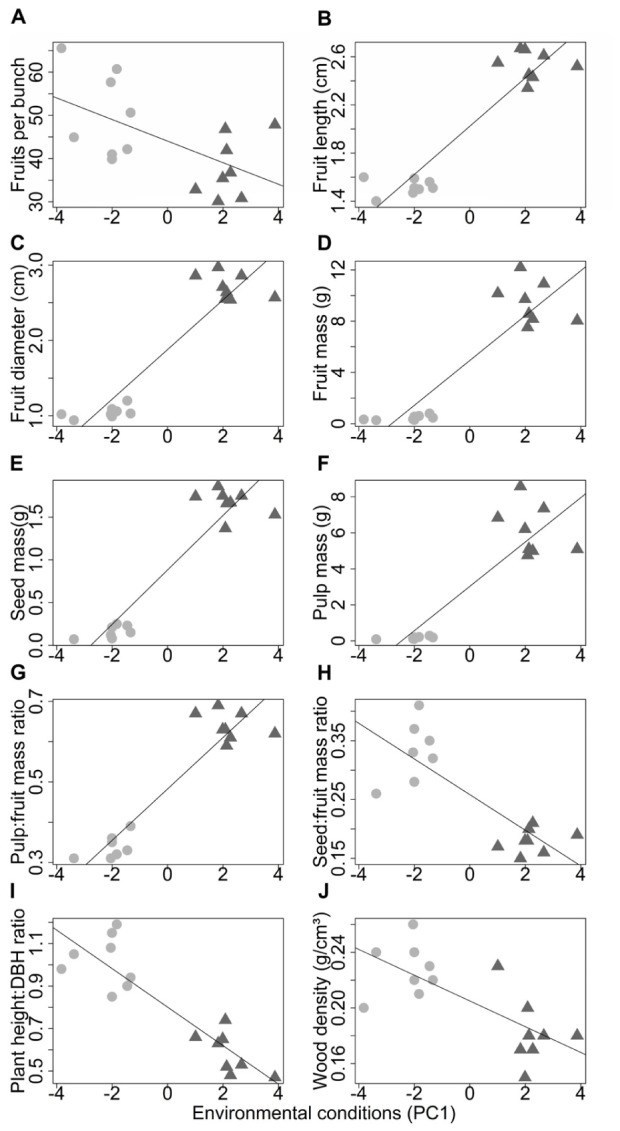
Relations between the morphological characteristics and the environmental conditions in wild populations (circles) and domesticated populations (triangles) of *Pourouma cecropiifolia*. The *x*-axis represents the environmental conditions (axis 1 of the PCA; **Supplementary Figure S1**), where higher values indicate higher sand content and light availability typical of cultivated areas on the *terra-firme*. Lower values indicate higher soil fertility, and silt and clay contents typical of floodplain forests.

The environmental conditions had significant effects on all the morphological characteristics (**Table 3**). The mass and dimensions of fruits (**Figures 6B–D**), seeds (**Figure 6E**), pulp (**Figure 6F**), and pulp:fruit mass ratio (**Figure 6G**) increase in environments with higher available light and poorer sandier soils (**Supplementary Figure S4**). The number of fruits per bunch (**Figure 6A**), seed:fruit mass ratio (**Figure 6H**), plant height:DBH ratio (**Figure 6I**) and wood density (**Figure 6J**) increase in environments with less available light and more fertile silty soils (**Supplementary Figure S4**). Analyzing only those characteristics less associated with domestication, we found a significant increase in wood density due to the increase in potassium content (**Supplementary Figure S4**).

**Table 3:** Results of the simple regression analyses between morphological characteristics of *P. cecropiifolia* populations (wild and domesticated, *n* = 16) and environmental conditions (PC1).

Trait	Coefficient	*t*-value	*p*-value	Adjusted *r*^2^
Fruits per bunch	–0.044	–1.774	0.103	0.120
Fruit length (cm)	**0.004**	4.271	<0.001	0.535
Fruit diameter (cm)	**0.006**	4.027	0.001	0.504
Fruit mass (g)	**0.034**	4.050	0.001	0.507
Seed mass (g)	**0.006**	3.927	0.002	0.507
Pulp mass (g)	**0.022**	3.704	0.003	0.476
Pulp:fruit mass ratio	**0.001**	3.945	0.002	0.510
Seed:fruit mass ratio	**–0.000**	–3.244	0.006	0.405
Plant height:DBH ratio (m/cm)	**–0.001**	–2.587	0.022	0.275
Wood density (g/cm^3^)	**–0.000**	–2.625	0.020	0.282

*The values of the coefficients in bold indicate that the relationship between the morphological characteristic and the environmental conditions is significant (*p* < 0.05).*

## Discussion

Using the traditional morphometric approach of domestication studies, we found higher mean values and greater variability in the dimensions and mass characteristics of the fruit in domesticated populations. The vegetative characteristics also varied, but to a lesser extent than the fruit characteristics. The domesticated populations showed lower values of plant height:DBH ratio and wood density than wild populations. Using an ecological approach, we found marked changes in the seed:fruit allometric relation. The domesticated fruits have a lower proportion of seed mass than the wild fruits of the same species and the fruits of 74 species of non-domesticated plants with the same fruit type (drupe). In addition, we observed that the morphological characteristics evaluated in *P. cecropiifolia* are influenced by variations in soil and light conditions. However, it is not easy to dissociate the environmental effect from the domestication effect, because farmers also created the cultivated landscapes.

### Morphological Characteristics in Domesticated Versus Wild Populations

The increase in dimensions and mass of fruits and seeds are the characteristics most modified during the domestication process of fruit species (Meyer et al., 2012). Domestication in fruit trees selects extreme phenotypes for size and mass of fruits, and eliminates phenotypes that differ from the preferred phenotype, reducing their frequencies in domesticated populations (Zohary, 2004). In *P. cecropiifolia*, humans selected for fruits with larger sizes and larger masses than those found in wild populations, in which large fruits are not favored by natural selection (McCouch, 2004). Dispersers of wild *P. cecropiifolia* in the forest are usually small-sized primates, such as *Saguinus mystax, S. fuscicollis* (Knogge and Heymann, 2003), *Callimico goeldii, Saguinus labiatus* (Porter, 2001) and *Cebus apella* (Gómez-Posada, 2012). Smaller fruits may provide an advantage over large fruits for dispersal by small-sized primates, because they can be more easily removed, transported and dispersed (Tanksley, 2004). On the other hand, in domesticated plants, the larger fruit size does not have an adverse effect, because humans guarantee the seeds’ dispersal and the seedlings’ establishment.

The number of fruits per bunch was smaller in domesticated populations than in wild populations. This negative correlation between number and size is common in fruit trees (Browning, 1985), due to the reallocation of photoassimilates to fruit size, which demands a decrease in the number of fruits per bunch for biomechanical reasons.

Within the domesticated populations we found geographic variation for fruit size, where a group of populations on the western side of the sample area had larger fruit than a group of populations to the east. This finding supports Clement’s (1989) proposal of a larger-fruited landrace close to the triple frontier (Brazil, Colombia, and Peru). Whether this extends as far as Iquitos, Peru, where Ducke (1946) commented on the abundance and popularity of *P. cecropiifolia*, remains to be investigated.

The lower mean values found in plant height:DBH ratio and in wood density can be explained by changes in edaphic and light conditions, which will be better detailed later in the specific section where the environmental effects on the morphological characteristics will be discussed. However, it is also possible that domestication generated a reduction in the height, diameter and wood density of the trees, due to the reallocation of photoassimilates to the harvestable product, changing the ratio between the biomass of the harvestable product (the fruits and seeds in the case of *P. cecropiifolia*) and the total plant biomass (Li et al., 2012). This ratio is called the ‘harvest index,’ and a negative correlation with plant height is common in many annual crops, such as rice (Li et al., 2012) and sorghum (Can and Yoshida, 1999) due to greater translocation of photoassimilates from the vegetative tissues to grains (Zou et al., 2003). For tree crops, this negative correlation is also expected (Cannell, 1985).

### Is the Variability in Characteristics Under Human Selection Higher?

In addition to the morphological differences between wild and domesticated populations that characterize the domestication syndrome, phenotypic variability is also expected to be greater in useful parts (Pickersgill, 2007). This expectation was observed in domesticated populations of banana (Li et al., 2013), peach palm (Clement, 1988) and tomato (Tanksley, 2004). Although genetic variability generally decreases in domesticated populations, phenotypic variability of selected parts may increase with domestication (McCouch, 2004; Purugganan and Fuller, 2009) due to dispersal and diversification after initial domestication (Meyer and Purugganan, 2013). During the dispersal process, the genetic material under selection is shared and disseminated among different human groups with cultural peculiarities, which may have, for example, different food preferences. This may also promote diversity in domestication syndromes (Milla et al., 2015). In the case of domesticated *P. cecropiifolia* populations, the Ticuna report fruits with more fibrous pulp and others whose pulp has higher water content, thus generating large variation in pulp and fruit mass. However, the Ticuna also report that they seldom select for the juicier pulp, as the fruits “explode” when they fall on the ground, a common occurrence during harvesting, and are unfit for transport or sale.

### Alterations in the Seed Mass:Fruit Mass Allometry

Humans selected *P. cecropiifolia* plants to have larger fruits. In response to selection to increase fruit mass, a faster increase in pulp mass than in seed mass is expected, resulting in fruit with a higher relative proportion of pulp (Martinez et al., 2007; Chen et al., 2010). The increase in pulp mass in *P. cecropiifolia* is mainly due to the increase in carbohydrates (fibers, cell walls, and starch) (Lopes et al., 1999). Due to the correlation among fruit components, the increase in fruit mass also leads to an increase in seed mass. However, the seeds of domesticated *P. cecropiifoli*a do not follow the same tendency of increase observed in wild plants (MS ∼ MF^0.89^; Niklas, 1994). Seeds contain proteins and oils, which are energetically more “expensive” than carbohydrates (Lopes et al., 1999). Therefore, it is likely that seed mass does not increase in the same proportion as fruit mass in domesticated plants, because the highest selective pressure is on the fruit, which responds with more carbohydrates, and not on the seed, which needs to retain a balance of proteins, carbohydrates, and oils to guarantee good germination.

### The Effects of Environmental Conditions and Their Relations With Domestication

Contrary to expectations for the variation in dimensions and mass of fruits and their components in response to edaphic conditions (Janick and Paull, 2008), we found fruits with larger masses and dimensions, and larger masses of seed and pulp in the domesticated populations, which occur in soils 16x poorer in nutrients and 63% sandier than floodplain soils, where wild populations occur. Having larger fruits with larger seeds and pulp mass in poorer and sandier soils is an indication that the domesticated phenotype is mainly a result of alterations in the genotype resulting from domestication, considering that for *P. cecropiifolia* the fruit characteristics are the most important for humans and are under direct human selection. The increase of fruit dimensions may have been due to the preferential selection of trees with large fruits and the elimination of trees with small fruits (Zohary, 2004). Considering that the size of the plant affects biomass allocation (Milla and Matesanz, 2017) and considering that the high light availability of the cultivated environment where the domesticated populations of *P. cecropiifolia* grow generates a decrease in the total size of the plant, it is possible that there is a trade-off that may have led to a lower allocation to vegetative parts and a higher allocation to reproductive organs, as is typical of changes in harvest index (Li et al., 2012).

**FIGURE 7:**
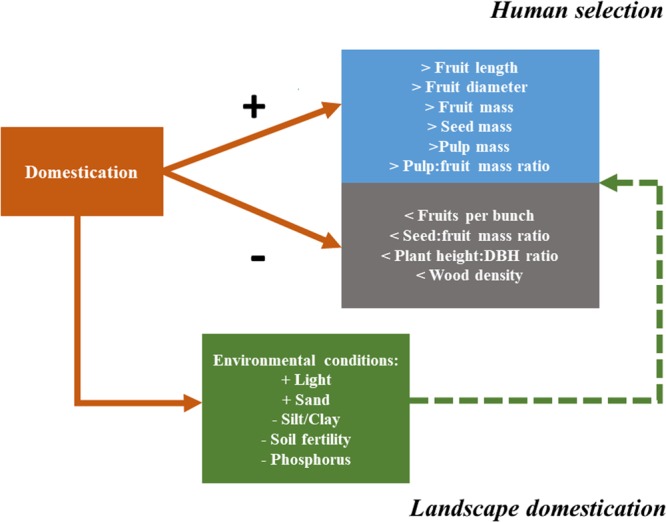
Diagram showing how domestication can influence plant phenotype via direct selection on plant characteristics (human selection) or via modification of environmental conditions (landscape domestication). The continuous arrows represent the direct effects of domestication by human selection on *Pourouma cecropiifolia* morphological characteristics. The dashed arrow represents the indirect effect of domestication, promoted by human management of environmental conditions (landscape domestication).

The subtle variations in vegetative characteristics suggests that they are not under direct selection. These changes are possibly results of changes in the environmental conditions caused by human management, an indirect effect of domestication (Harlan, 1992; Zohary, 2004) (**Figure 7**). Due to high light availability in cultivated landscapes, the plants of domesticated populations invested less in growth in height, because the competition with other plants for light is smaller when compared to individuals in forest landscapes that have smaller canopy openings (Cannell, 1985). With the greater light availability in the cultivated environment, domesticated plants possibly grow faster than wild plants, and consequently have lower wood density (Poorter, 1999; Poorter et al., 2008). The plant height:DBH in domesticated plants is also affected by the sandier soils of the cultivated areas. Sandy soils maintain less water and nutrients due to their lower surface areas (Tarboton, 2003), which affects water supply in dry seasons.

Under natural conditions, wild *P. cecropiifolia* plants produce proportionately larger seeds due to the difficult conditions of establishment in the shaded understory, as observed in other forest species (Salisbury, 1974; Westoby et al., 1996). In cultivated landscapes where the *P. cecropiifolia* domesticated plants grow, the low fertility of cultivated soils (Westoby et al., 1990; Hammond and Brown, 1995) and the high light availability promote reduction of seed mass (Eriksson et al., 2000; Moles et al., 2005). In contrast, human selection for larger fruits and the positive correlation between fruit mass and seed mass promote an increase in seed mass in the domesticated plants. Therefore, the combined but contrasting environmental effects and human selection lead domesticated populations to have a proportionately smaller increase in seed mass than wild populations.

By evaluating individually the variables not correlated with landscape domestication, it was possible to observe which morphological changes are effectively responding to environmental variations. The positive correlation between wood density and potassium content in the soil, and the existence of one domesticated population with wood density similar to the wood density of the wild populations, in a site with a high potassium content, shows the environmental effect on the phenotypic plasticity of the *P. cecropiifolia* individuals. This suggests that, although the effect of environmental conditions in cultivated landscapes can be superimposed on the domestication effect, we cannot ignore the plastic capacity of individuals in explaining the morphological variations of plants under human selection.

Due to the difficulty in dissociating the effect of environmental conditions from the effect of human selection, we suggest that reciprocal transplant experiments of domesticated plants to uncultivated landscapes and wild plants to cultivated landscapes will be needed to effectively differentiate domestication effects from environmental effects. In addition, we consider that in future studies it will be necessary to evaluate experimentally the effect of luminosity on fruit mass, seed mass and in seed:fruit allometry considering the high light availability in cultivated landscapes and because it is expected that there is a positive correlation between light intensity and fruit mass (Moles et al., 2005; Janick and Paull, 2008), but a negative correlation with seed mass (Eriksson et al., 2000; Moles et al., 2005).

In this study, the domesticated phenotype is a result of a combination of human selection and environmental conditions in the sites where the plants are cultivated. We observed strong environmental modification created by humans in cultivated landscapes that is exacerbated by the fact that wild populations occur in flooded areas, while domesticated populations occur in upland areas. These changes in environmental conditions between natural and cultivated sites, in addition to genetic selection by humans, promoted the phenotypic changes in domesticated populations. However, the forces of genetic selection through human management and of natural selection through environmental conditions are intrinsically mixed and discriminating the magnitude of each component, and the environment by genotype interaction, requires a well-designed common garden experiment (Falconer and Mackay, 1996).

## Conclusion

Addressing ecological aspects in plant domestication studies provides us with a more integrated understanding about the evolution of cultivated plants, because the domesticated phenotypes are the result of the combined effects of human selection and natural selection on plant populations. We quantified modifications in numerous components of the domestication syndrome of *P. cecropiifolia* populations in Western Amazonia. The domesticated plants presented substantial changes in the morphology of their fruits and seeds, and more subtle changes in vegetative characteristics. The combined effect of natural selection and human selection modified the expected pattern in the allometric relations between seed mass and fruit mass, due to the contrasting effects of environmental filters, which promote seed size reduction, and human selection, which promotes seed size increase. The strong correlation between domestication and environmental conditions due to changes in the landscape generated by human management made it difficult to separate environmental effects from human selection effects. The evaluation of the effects of environmental conditions and of human selection and management in cultivated landscapes are important for a better understanding of the domestication syndrome. We suggest that the allometric differences between fruits and seeds of wild and domesticated plants can be used in future studies, as an additional parameter of the domestication syndrome.

## Data Accessibility

Data for this paper have been archived in figshare: https://figshare.com/articles/data_Pcecropiifolia_xlsx/5306380.

## Author Contributions

HP: contributed to conception and design of the study, was responsible for field collections and laboratory analyses, conducted the statistical analyses, contributed to the interpretation of the results, wrote the first version of the manuscript, and approved the final version to be published. CC: contributed to conception and design of the study and statistical analyses, participated substantially in the interpretation of the results and drafting of the manuscript, and approved the final version to be published. JS: contributed to conception and design of the study, participated substantially in the statistical analyses, interpretation of the results, and drafting of the manuscript, and approved the final version to be published.

## Conflict of Interest Statement

The authors declare that the research was conducted in the absence of any commercial or financial relationships that could be construed as a potential conflict of interest. The handling editor is currently co-organizing a Research Topic with one of the authors CC, and confirms the absence of any other collaboration.

## Supplementary Material

FIGURE S1 Principal components analysis of the environmental variables at each cultivated and natural site where *Pourouma cecropiifolia* was collected for this study.

FIGURE S2 Correlation matrix among the morphological characteristics of *Pourouma cecropiifolia*.

FIGURE S3 Correlation matrix between domestication status of *Pourouma cecropiifolia* and the environmental variables and among the environmental variables.

FIGURE S4 Correlation matrix among the morphological characteristics of *Pourouma cecropiifolia*, among the environmental variables and between the morphological characteristics and the environmental variables.

TABLE S1 Descriptivie statistics at each site for all 10 morphological characteristics analyzed of *Pourouma cecropiifolia*.

TABLE S2 Results of ANOVA between the wild and domesticated groups of *Pourouma cecropiifolia* for each characteristic analyzed.

TABLE S3 Morphological caracteristics of *Pourouma cecropiifolia* and environmental variables by population, including the values of pH, soil nutrients, soil texture and Crown Illumination Index.
