# Supplementary figures and images for: The Domestication of the Amazon Tree Grape (Pourouma cecropiifolia) Under an Ecological Lens
Source: Front Plant Sci. 2018 Mar 14;9:203. doi: 10.3389/fpls.2018.00203 (PMC5861524; doi:10.3389/fpls.2018.00203)

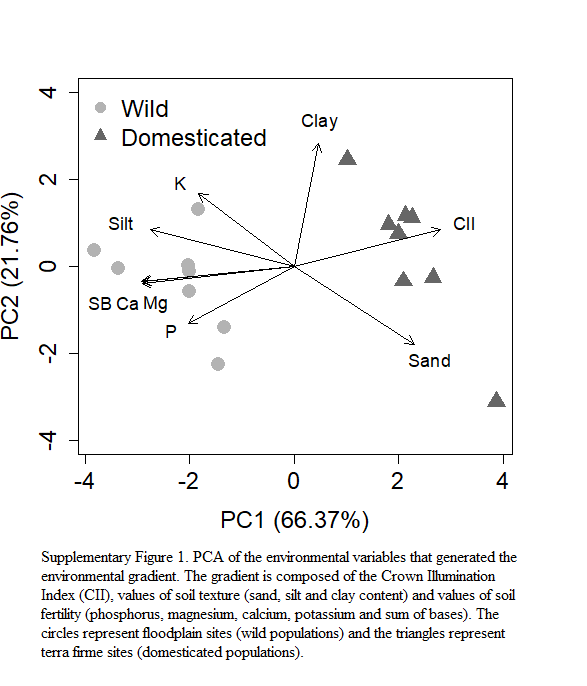

Supplement: FIGURE S1 — Principal components analysis of the environmental variables at each cultivated and natural site where Pourouma cecropiifolia was collected for this study. [file Image_1.TIFF]

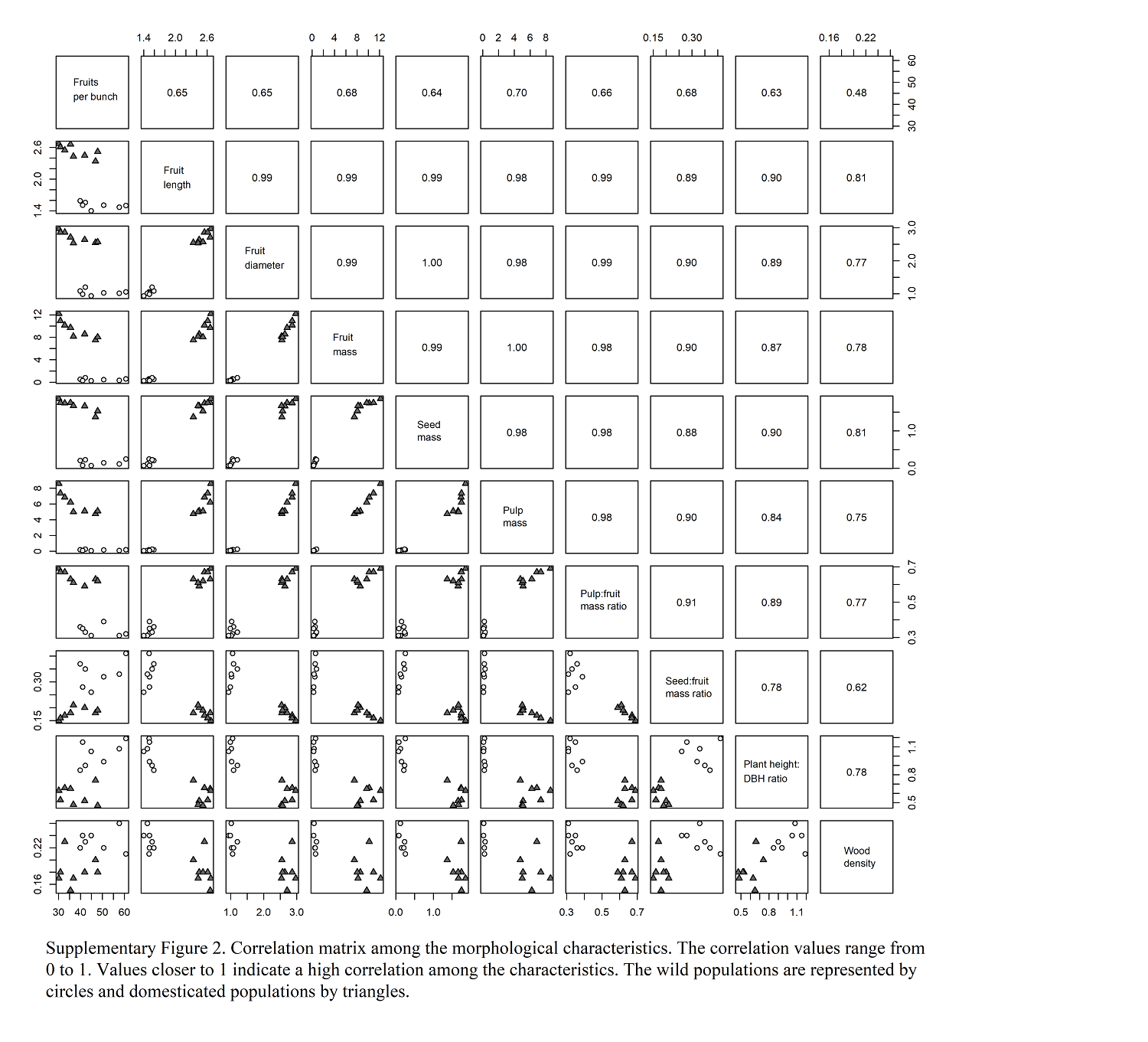

Supplement: FIGURE S2 — Correlation matrix among the morphological characteristics of Pourouma cecropiifolia. [file Image_2.TIFF]

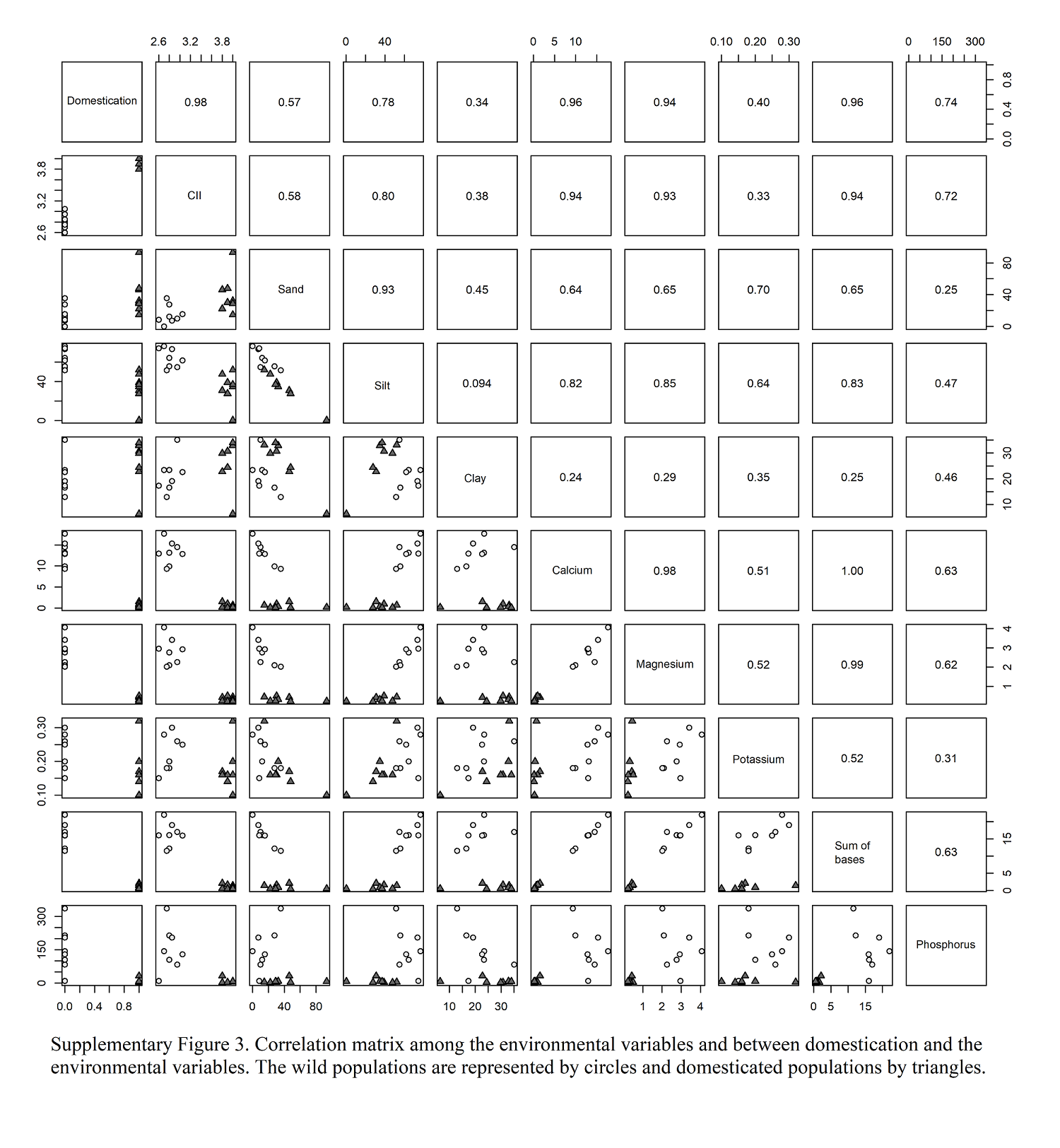

Supplement: FIGURE S3 — Correlation matrix between domestication status of Pourouma cecropiifolia and the environmental variables and among the environmental variables. [file Image_3.TIFF]

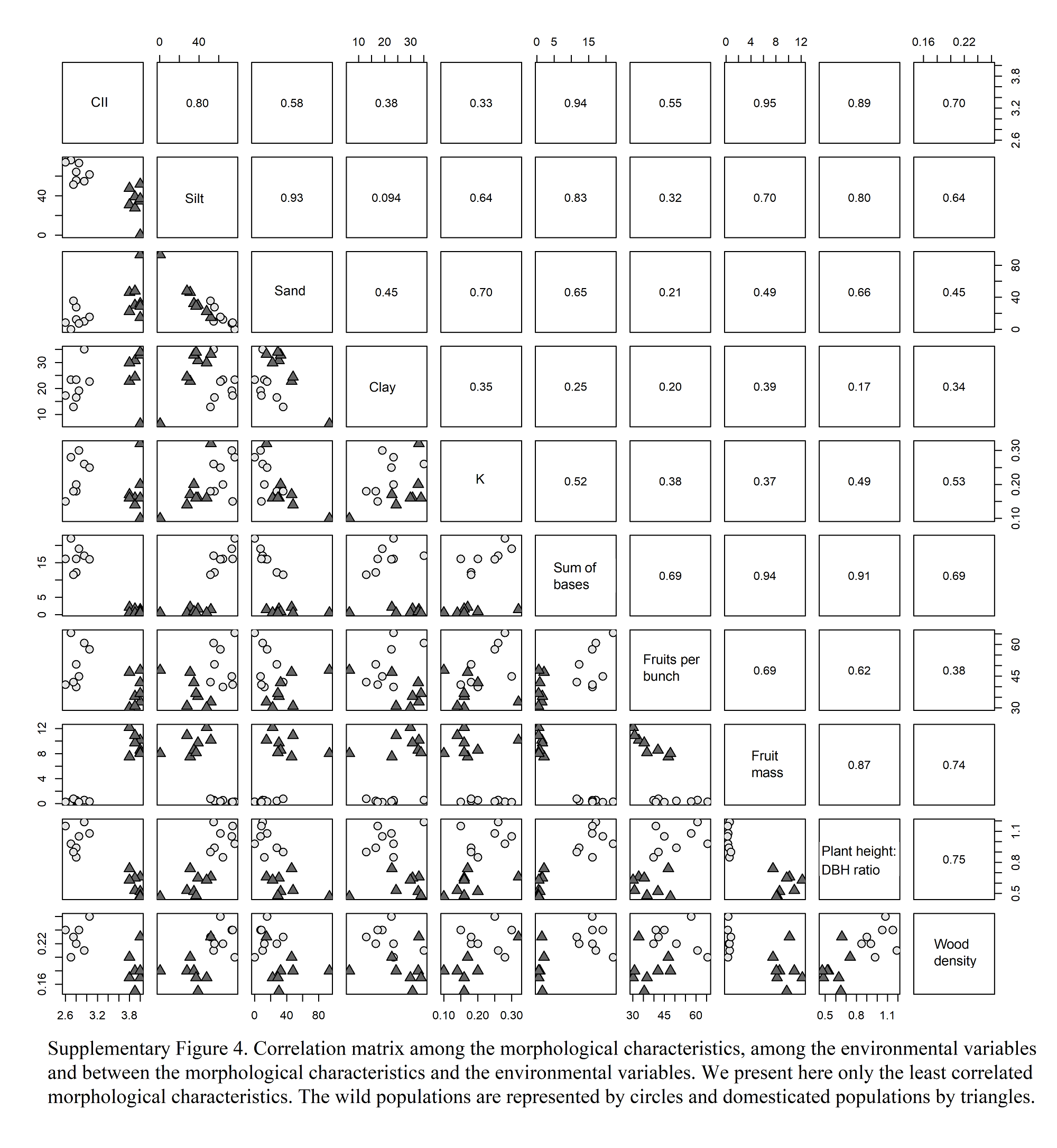

Supplement: FIGURE S4 — Correlation matrix among the morphological characteristics of Pourouma cecropiifolia, among the environmental variables and between the morphological characteristics and the environmental variables. [file Image_4.TIFF]
