# Supplementary material for: The Domestication of the Amazon Tree Grape (Pourouma cecropiifolia) Under an Ecological Lens
Source: Front Plant Sci. 2018 Mar 14;9:203. doi: 10.3389/fpls.2018.00203 (PMC5861524; doi:10.3389/fpls.2018.00203)
Supplement: TABLE S2 — Results of ANOVA between the wild and domesticated groups of Pourouma cecropiifolia for each characteristic analyzed. [file Table_2.DOCX]

Supplementary Material

**The domestication of the Amazon tree grape (*Pourouma cecropiifolia*) under an ecological lens**

**Hermísia C. Pedrosa*, Charles R. Clement and Juliana Schietti**

*** Correspondence:** Corresponding Author: [hermisia.pedrosa@gmail.com](mailto:hermisia.pedrosa@gmail.com)

Supplementary Table 2. ANOVA table between the wild and domesticated group for each trait, with the degrees of freedom of the effects and of the residuals (in parentheses), error term, F-values and p-values

| *Trait* | *df* | *Error term* | *F-value* | *p-value* |
| --- | --- | --- | --- | --- |
| Fruits per bunch | 1(158) | 12.931 | 37.25 | <0.001 |
| Fruit lenght (cm) | 1(158) | 0.157 | 1699.00 | <0.001 |
| Fruit diameter (cm) | 1(158) | 0.194 | 2965.00 | <0.001 |
| Fruit mass (g) | 1(156) | 1.554 | 1312.00 | <0.001 |
| Seed mass (g) | 1(146) | 0.215 | 1805.00 | <0.001 |
| Pulp mass (g) | 1(147) | 1.279 | 802.10 | <0.001 |
| Pulp:fruit mass ratio | 1(147) | 0.048 | 1464.00 | <0.001 |
| Seed:fruit mass ratio | 1(147) | 0.054 | 287.20 | <0.001 |
| Plant height:DBH ratio (m/cm) | 1(158) | 0.276 | 98.97 | <0.001 |
| Wood density (g/cm³) | 1(147) | 0.047 | 31.40 | <0.001 |
|  |  |  |  |  |
