# Supplementary material for: The Domestication of the Amazon Tree Grape (Pourouma cecropiifolia) Under an Ecological Lens
Source: Front Plant Sci. 2018 Mar 14;9:203. doi: 10.3389/fpls.2018.00203 (PMC5861524; doi:10.3389/fpls.2018.00203)
Supplement: TABLE S3 — Morphological caracteristics of Pourouma cecropiifolia and environmental variables by population, including the values of pH, soil nutrients, soil texture and Crown Illumination Index. [file Table_3.DOCX]

Supplementary Material

**The domestication of the Amazon tree grape (*Pourouma cecropiifolia*) under an ecological lens**

**Hermísia C. Pedrosa*, Charles R. Clement and Juliana Schietti**

*** Correspondence:** Corresponding Author: hermisia.pedrosa@gmail.com

Supplementary Table 3. Mean values of environment variables and morphological characteristics by population.

| **Population** | **Group** | **Crow Illumitation Index (CII)** | **Sand (%)** | **Clay (%)** | | **Silt (%)** | **Ca (cmolc.kg)** | **Mg**  **(cmolc.kg)** | **K (cmolc.kg)** | **P**  **(mg.kg)** | **SB** |
| --- | --- | --- | --- | --- | --- | --- | --- | --- | --- | --- | --- |
| 1 | Wild | 2.70 | 0.16 | 23.42 | 76.42 | | 17.71 | 4.07 | 0.28 | 143.56 | 22.06 |
| 2 | Wild | 2.95 | 10.12 | 35.10 | 54.78 | | 14.52 | 2.26 | 0.26 | 83.12 | 17.04 |
| 3 | Wild | 2.80 | 27.77 | 16.59 | 55.64 | | 9.93 | 2.10 | 0.18 | 213.57 | 12.21 |
| 4 | Wild | 2.80 | 12.33 | 23.38 | 64.29 | | 13.15 | 2.76 | 0.20 | 104.49 | 16.10 |
| 5 | Wild | 2.75 | 35.52 | 12.95 | 51.53 | | 9.33 | 2.02 | 0.18 | 335.18 | 11.53 |
| 6 | Wild | 3.05 | 15.69 | 22.62 | 61.69 | | 12.85 | 2.92 | 0.25 | 129.55 | 16.01 |
| 7 | Wild | 2.85 | 7.48 | 19.14 | 73.38 | | 15.34 | 3.41 | 0.30 | 204.73 | 19.05 |
| 8 | Wild | 2.60 | 8.48 | 17.33 | 74.19 | | 12.97 | 2.96 | 0.15 | 9.29 | 16.08 |
| 9 | Domesticated | 4.00 | 14.88 | 33.12 | 52.00 | | 0.68 | 0.45 | 0.32 | 4.90 | 1.45 |
| 10 | Domesticated | 3.80 | 46.28 | 22.79 | 30.93 | | 1.52 | 0.43 | 0.17 | 32.27 | 2.11 |
| 11 | Domesticated | 3.90 | 30.28 | 30.72 | 39.00 | | 0.97 | 0.50 | 0.16 | 3.91 | 1.63 |
| 12 | Domesticated | 3.80 | 22.41 | 29.86 | 47.73 | | 0.15 | 0.24 | 0.16 | 2.47 | 0.55 |
| 13 | Domesticated | 3.90 | 47.91 | 24.41 | 27.68 | | 0.10 | 0.22 | 0.14 | 3.43 | 0.47 |
| 14 | Domesticated | 4.00 | 32.40 | 32.87 | 34.73 | | 0.36 | 0.32 | 0.20 | 8.55 | 0.88 |
| 15 | Domesticated | 4.00 | 28.98 | 33.99 | 37.03 | | 0.10 | 0.24 | 0.16 | 7.22 | 0.50 |
| 16 | Domesticated | 4.00 | 93.23 | 6.41 | 0.36 | | 0.21 | 0.23 | 0.10 | 7.26 | 0.54 |

Supplementary Table 3. (Continued) Mean values of environment variables and morphological characteristics by population.

| **Population** | **Group** | **Fruits per bunch** | **Fruit**  **length (cm)** | **Fruit**  **diameter (cm)** | **Fruit mass (g)** | **Seed mass (g)** | **Pulp mass (g)** | **Pulp:fruit mass ratio** | **Seed: fruit mass ratio** | **Height (m)** | **DBH (cm)** | **Plant height:**  **DBH ratio (m/cm)** | **Wood density (g/cm³)** |
| --- | --- | --- | --- | --- | --- | --- | --- | --- | --- | --- | --- | --- | --- |
| 1 | Wild | 65.52 | 1.60 | 1.02 | 0.32 | NA | NA | NA | NA | 16.43 | 18.49 | 0.98 | 0.20 |
| 2 | Wild | 60.70 | 1.50 | 1.06 | 0.60 | 0.25 | 0.21 | 0.32 | 0.41 | 17.11 | 16.27 | 1.19 | 0.21 |
| 3 | Wild | 50.62 | 1.51 | 1.03 | 0.46 | 0.15 | 0.18 | 0.39 | 0.32 | 17.10 | 21.17 | 0.94 | 0.22 |
| 4 | Wild | 39.84 | 1.59 | 1.09 | 0.54 | 0.21 | 0.19 | 0.36 | 0.37 | 19.37 | 24.43 | 0.85 | 0.22 |
| 5 | Wild | 42.16 | 1.56 | 1.20 | 0.79 | 0.23 | 0.28 | 0.33 | 0.35 | 15.10 | 20.36 | 0.90 | 0.23 |
| 6 | Wild | 57.66 | 1.47 | 1.02 | 0.34 | 0.12 | 0.11 | 0.31 | 0.33 | 21.22 | 21.55 | 1.08 | 0.26 |
| 7 | Wild | 44.92 | 1.40 | 0.94 | 0.26 | 0.07 | 0.08 | 0.31 | 0.26 | 17.86 | 18.45 | 1.05 | 0.24 |
| 8 | Wild | 40.98 | 1.51 | 0.99 | 0.28 | 0.08 | 0.10 | 0.35 | 0.28 | 18.05 | 17.14 | 1.15 | 0.24 |
| 9 | Domesticated | 32.82 | 2.55 | 2.86 | 10.16 | 1.74 | 6.84 | 0.67 | 0.17 | 9.36 | 16.52 | 0.65 | 0.23 |
| 10 | Domesticated | 46.84 | 2.34 | 2.55 | 7.51 | 1.37 | 4.75 | 0.63 | 0.18 | 9.42 | 14.42 | 0.74 | 0.20 |
| 11 | Domesticated | 35.44 | 2.66 | 2.71 | 9.72 | 1.75 | 6.21 | 0.63 | 0.18 | 8.58 | 14.06 | 0.65 | 0.15 |
| 12 | Domesticated | 30.12 | 2.67 | 2.97 | 12.20 | 1.86 | 8.58 | 0.69 | 0.15 | 7.84 | 14.76 | 0.63 | 0.17 |
| 13 | Domesticated | 30.82 | 2.61 | 2.86 | 10.92 | 1.75 | 7.35 | 0.67 | 0.16 | 11.42 | 24.01 | 0.53 | 0.18 |
| 14 | Domesticated | 41.92 | 2.45 | 2.64 | 8.57 | 1.66 | 5.10 | 0.59 | 0.20 | 8.05 | 16.94 | 0.52 | 0.17 |
| 15 | Domesticated | 36.76 | 2.43 | 2.54 | 8.17 | 1.67 | 4.99 | 0.61 | 0.21 | 7.51 | 17.18 | 0.48 | 30.18 |
| 16 | Domesticated | 47.84 | 2.52 | 2.57 | 8.04 | 1.53 | 5.09 | 0.62 | 0.19 | 9.35 | 20.96 | 0.47 | 3.38 |
